# A commentary on ‘Early plasma proteomic biomarkers and prediction model of acute respiratory distress syndrome after cardiopulmonary bypass: a prospective nested cohort study’

**DOI:** 10.1097/JS9.0000000000001477

**Published:** 2024-05-23

**Authors:** Xin Zhu, Peng Liu

**Affiliations:** a Department of Anesthesiology, The First Affiliation hospital of Dalian Medical University; b Department of Heart Intensive Care Unit, The first Affiliated Hospital of Dalian Medical University, Dalian, Liaoning Province, People’s Republic of China

*Dear Editor,*

Cardiopulmonary bypass (CPB) may cause systemic inflammation, resulting in acute lung injury (ALI), including acute respiratory distress syndrome (ARDS), a potentially fatal complication in patients following heart surgery^1,2^. Wang *et al*.^3^ conducted three prospective nested cohort studies of all consecutive patients undergoing cardiac surgery with CPB. It not only demonstrates that NPC intracellular cholesterol transporter 2 (NPC2), cathepsin L (CTSL), and plasma thioredoxin domain containing 5 (TXNDC5) may be predictive biomarkers for ARDS following CPB surgery, but it also constructed and validated a parsimonious model based on proteomic and clinical aspects.

Cohort 1 included 150 patients between April 2021 and July 2021 for the goal of discovery-based biomarker screening. A total of 709 proteins were found; in cohort 1, the differences in protein levels between ARDS cases and controls at T1, T2, and T3 were 9, 29, and 35, respectively. The top four protein features – NPC2, CD56, TXNDC5, and CTSL – with increased percentages of more than 10% were regarded as potential biomarkers for the prediction of CPB-ARDS, according to machine learning-based inference of biomarkers. The association between CPB-ARDS and TXNDC5, CTSL, and NPC2 was further demonstrated by the Spearman correlation coefficient values and the validation of the biomarker alterations’ expressions. Furthermore, the lung tissue of LIRI (lung ischemia reperfusion injury) mice showed a notable elevation of the significantly overexpressed TXNDC5 and CTSL proteins found in the plasma of patients with CPB-ARDS.

Cohort 2 included 375 patients between August 2021 and July 2022 for the goal of validation strategies. Increased levels of TXNDC5, CTSL, and NPC2 at T2 were noted in CPB-ARDS patients after the quantitative validation of multiple prognostic proteins in cohort 2. The data also shed light on the dynamic online predictive nomogram that was created. Cohort 3 included 124 patients from January 2023 to March 2023 with the purpose of evaluating the prediction mode’s external validity. Based on two clinical risk variables and three proteins, a dynamic online predictive nomogram was created, and it performed exceptionally well (precision: 83.33%, sensitivity: 93.33%, specificity: 61.16%, and F1 score: 85.05%). There were several limitations, though. First, because this was a single-center study, its generalizability was limited. Secondly, uncontrolled confounders may have affected the results. Lastly, there are certain flaws in the model used to predict serious pulmonary problems^4^.

In order to predict CPB-ARDS risk individually, this work found a number of novel predictive biomarkers and created and validated a useful prediction method that used biomarker and clinical component combinations. After major surgery, the plasma TXNDC5, CTSL, and NPC2 levels may help identify patients who need more intensive therapy and closer monitoring to prevent ARDS. This problem has far-reaching clinical ramifications. This innovative strategy offers insightful direction and motivation for the ongoing advancement of this field of study. Further studies are required on a number of fronts in the context of ARDS following heart surgery (risk factors, diagnosis, therapy possibilities)^5^. A more thorough identification of the risk factors is required, as lung damage prevention seems to be of the utmost relevance. Extracorporeal methods may be a viable option in expert hands for individuals who develop ARDS after heart surgery. NMBAs (neuromuscular blocking agents), prone positioning, and iNO (inhaled nitric oxide) can be used on an individual basis, but vigorous lung recruitment and oscillatory breathing should be avoided.

## Ethical approval

Not applicable.

## Consent

Not applicable.

## Author contribution

X.Z.: study concept or design, data collection, data analysis or interpretation, and writing the paper; P.L.: study concept or design, data collection, data analysis or interpretation, writing and revising the paper.

## Conflicts of interest disclosure

The authors declares no conflicts of interest.

## Research registration unique identifying number (UIN)

Not applicable.

## Guarantor

Peng Liu.

## Data availability statement

This manuscript is a comment. Don’t need a Data availability statement. However, all the data from the current study are publicly available.

## Provenance and peer review

Not applicable.
